# Modeling and development of technology for smelting a complex alloy (ligature) Fe-Si-Mn-Al from manganese-containing briquettes and high-ash coals

**DOI:** 10.1038/s41598-024-57529-6

**Published:** 2024-03-29

**Authors:** Assylbek Nurumgaliyev, Talgat Zhuniskaliyev, Viktor Shevko, Yerbol Mukhambetgaliyev, Bauyrzhan Kelamanov, Yerbol Kuatbay, Alexandra Badikova, Gauhar Yerekeyeva, Irina Volokitina

**Affiliations:** 1 Karaganda Industrial University, Temirtau, Kazakhstan; 2 South Kazakhstan University Named After M. Auezov, Shymkent, Kazakhstan; 3 Chemical and Metallurgical Institute Named After Zh. Abishev, Karaganda, Kazakhstan; 4 Aktobe Industrial University Named After K. Zhubanov, Aktobe, Kazakhstan

**Keywords:** Manganese ore, Coal, Ore-thermal electric smelting, Thermodynamic modeling, Rotatable planning, Complex ligature, Materials for energy and catalysis, Theory and computation, Process chemistry

## Abstract

Investigation of the possibility of obtaining a complex master alloy used in the deoxidation of steel, smelted from substandard manganese-containing materials, briquettes, and high-ash coals in ore-thermal electric furnaces. Thermodynamic modeling was carried out using the HSC Chemistry software package to determine the optimal process parameters using a second-order rotatable plan (Box-Hunter plan). Thermodynamic modeling improves the understanding of physical and chemical processes, allows making predictions about the behavior of the system under various conditions, optimizing processes and saving time and resources necessary for experiments. Electric smelting of the briquette was carried out with coal and quartzite (to adjust the chemical composition and neutralize residual carbon) in an ore-thermal electric furnace with a power of up to 150 kV*A. The influence of temperature on the equilibrium distribution of silicon, manganese, and aluminum in the «briquette-coal-quartzite» system, the degree of transition of silicon and manganese into a complex ligature and the content of these metals in the alloy are determined by the method of thermodynamic modeling. As a result of experiments on ore-thermal electric smelting of a briquette with high-ash coal, a complex ligature was obtained with an average content of 45.92–53.11% silicon, 27.72–34.81% manganese and 5.60–6.91% aluminum.

## Introduction

At present, the world steel production is about 2 billion tons per year. In 2021, Kazakhstan produced 4.4 million tons of steel, increasing steel production by 15% more comparison since 2020. The competitiveness of steel produced largely depends on reducing its cost. In this regard, one of the priority areas for the development of ferrous metallurgy is the creation and production of new multi-purpose materials used in the processing of steel based on natural raw materials and man-made waste^1,2^.

It is known that a significant improvement in the quality of steel can be achieved with the use of complex ligatures containing manganese, silicon and aluminum, which at the stage of deoxidation contribute to a deeper purification of steel from oxygen and non-metallic inclusions^3–6^.

In addition, the use of complex ligatures containing silicon, manganese and aluminum, obtained from substandard natural manganese-containing and carbon-containing raw materials, will significantly reduce the cost of the metal product and make it more competitive. In particular for ferroalloys containing silicon, manganese and aluminum.

One of the promising areasto address this. The problem is the organization of the production of complex ligatures based on substandard manganese Kazakh ores and high-ash coals^3,7–12^.

To research and develop the technology for smelting complex master alloys using a multicomponent charge containing silicon, manganese, aluminum and iron, it is necessary to conduct complex and time-consuming experiments under high-temperature conditions that depend on several parameters of temperature, pressure, concentration (chemical and phase compositions, etc.). In this regard, in research and production practice, methods of thermodynamic modeling of physicochemical processes under high-temperature conditions are successfully used, where the distribution of elements and components is predicted, taking into account the phase transformations Me-Slag-Gas^13–15^.

In physical and chemical studies, when studying complex processes occurring in a heterogeneous environment, with a certain degree of conventionality, two approaches can be distinguished. Thermodynamic, focused on solving the following problem: for a given initial composition of the system under study and fixed values of the equilibrium parameters (P-pressure, T-temperature), determine the equilibrium chemical composition of the reacting mixture and their amounts in the individual constituent phases and, based on this, evaluate the likely direction of the reaction^13–15^. There are kinetic and dynamic methods that make it possible to trace changes in time and space of the nonequilibrium component composition of the system depending on the rates of chemical reactions and the dynamics of reagents. Kinetics here means the study of physical and chemical processes in time, and dynamic in time and space. In this paper, when studying a new technology, only the thermodynamic approach is considered, one of the effective methods for predicting and environmental friendliness of the developed technology^16–18^.

The advantage of thermodynamic modeling (TDM) lies in its universal nature, which makes it possible to study systems of arbitrary chemical composition based on only reference information on the thermochemical and thermodynamic properties of individual substances. These properties are known for a wide range of chemical compounds in the gaseous, condensed and ionized state, at temperature range typical for most engineering applications^19–23^.

The use of TDM makes it possible to quantitatively model and predict the composition and properties of complex heterogeneous, multi-element, multi-phase systems in a wide range of temperatures and pressures, taking into account chemical and phase transformations^24–26^.

The purpose of this work is modeling, development and approbation of the process of obtaining complex ligature Fe-Si-Mn-Al from manganese-containing briquettes and high-ash coals using the HSC Chemistry software package.

**Figure 1:**
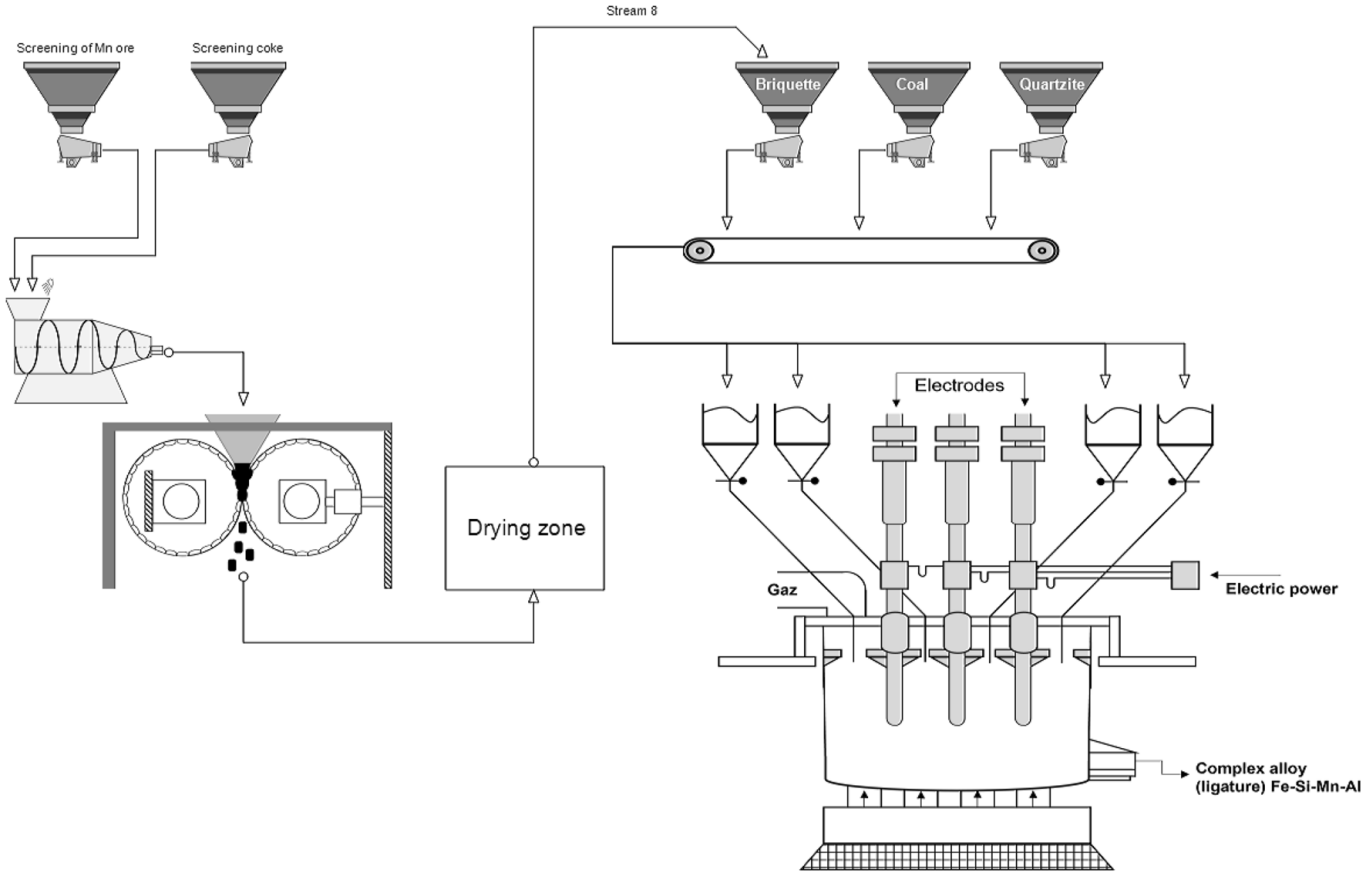
Technological scheme for the production of complex ligature Fe-Si-Mn-Al.

The proposed technological scheme for the production of complex master alloys is shown in the Fig. 1.

## Materials and methods

The theoretical substantiation of obtaining a complex ligature was carried out by us using thermodynamic modeling, and experimental confirmation and determination of the optimal parameters for the process of smelting a complex ligature was carried out on a laboratory ore-thermal electric furnace.

Thermodynamic modeling of the system was carried out using the HSC Chemistry software package (Outokumpu, Finland), based on the minimization of the Gibbs energy and variational principles of thermodynamics^27–33^. The software package was developed based on the ideology of the SGTE (Scientific Group Thermodata Europe) consortium. The SGTE consortium consists of research centers in Germany, Canada, France, Sweden, Great Britain and the USA that develop thermodynamic databases for inorganic and metallurgical systems and apply them to solve practical problems. The equilibrium composition of a multicomponent oxide and metal system was determined using the module of the Equilibrium Compositions software package in the temperature range of 500–2200 °C with a step of 100 °C and a gas phase pressure of 1 atm.

1 $${\text{G}}\left({\text{x}}\right)=\sum_{{\text{a}}=1}^{{\text{f}}}*\sum_{{\text{j}}=1}^{{\text{ia}}}{{\text{X}}}_{{\text{j}}}\left({{\text{C}}}_{{\text{j}}}+{\text{ln}}\left(\frac{{{\text{X}}}_{{\text{j}}}}{{{\text{X}}}_{{\text{a}}}}\right)+{\text{ln}}{\upgamma }_{{\text{j}}}\right)\to {\text{G}}{\left({\text{x}}\right)}_{min}$$

under restrictions in the form of a system of linear equations for the mass balance of matter:

2 $$\sum_{j=1}^{m}{a}_{i}j{X}_{j}={b}_{i}$$

and the normalization condition:

3 $$\sum_{j=1}^{La}{X}_{j}={X}_{a}$$

where f—is the total number of system phases; bi—is the total number of independent components in the system; i—is the mass of numbers showing the number of j-th independent components in the phase (α) of the system;—is the number of independent system components; Cj—is an empirical thermodynamic function; Xa—is the total number of moles of phase (a) in the system; $\frac{{X}_{j}}{{X}_{a}}$—mole fraction of the dependent component in phase (a); Γj—is the activity factor of the component.

The equilibrium parameters of a thermodynamic system are determined by solving the mathematical problem of finding an extremum, taking into account all restrictions, using the Lagrange functions and Newton's method of successive approximations.

When working with software complex HSC Chemistrythe initial information was presented in the form of a quantitative (kg) distribution of substances in the system under study. High-ash coal, manganese-containing briquette and quartzite were used as charge materials (Tables 1 and 2). Quartzite is used forneutralization of residual carbon andadjustment of the chemical composition in the charge mixture. The datasets generated and/or analysed during the current study are available in the Google Drive repository, https://share.kz/gqTQ. Then, in accordance with^34^, the equilibrium degree of the element (α, %) was determined from the interaction products. For this, calculations were carried out according to "Eq. (4)":4where, —mass of the element in the original system, kg; —mass of the element in the resulting product, kg. M—molecular weight of the initial substance in which the i-th element is located; —is the atomic mass of the i-th element; n—is the number of atoms of the i-th element in the initial substance; m—is the number of atoms of the i-th element in the final substance;

**Table 1:** Technical composition of charge materials.

Material	Ad	Vd	W	C
Coal «Saryadyr»	50.04	19.28	1.98	31.86
Briquette	63.01	14.94	5.58	16.47

*Where *Ad* ash content of coal (d on dry weight), *Vd* volatile components, *W* humidity.

**Table 2:** Chemical composition of charge materials.

Material	Fe_2_O_3_	SiO_2_	Al_2_O_3_	MnO_2_	CaO	MgO	TiO_2_	K_2_O	Na_2_O	P_2_O_5_	S	*LOI
Coal ash «Saryadyr»	5.79	66.36	20.7	–	2.64	3.46	1.01	–	–	0.035	0.005	–
Briquette	6.41	18.9	1.96	53.71	16.53	1.02	0.02	0.02	1.34	0.04	0.05	–
Quartzite	0.52	95.57	–	–	0.24	0.12	–	–	–	–	0.01	3.54

*Loss on ignition.

The calculation of the equilibrium degree of distribution of elements was carried out according to the methodology developed by scientists of the South Kazakhstan University named after. M. Auezov. When searching for the optimal conditions for the transition of the main elements of the complex ligature into the alloy, the method of research based on second-order rotatable plans (the Box-Hunter plan) was used with obtaining an adequate regression equation and constructing a geometric figure of the optimization parameters (the significance of the coefficients of the equation was determined by Student's criterion, the adequacy of the equation—according to Fisher's criterion), which has been described and implemented repeatedly^35–39^.

Table 3 shows a research planning matrix for obtaining complex ligatures from manganese briquettes, coal and quartzite. In all experiments, the mass of the briquette was constant and amounted to 100 kg. The blending was done based on 100 kg briquette:—80 kg coal and 39.38 kg quartzite. The ore recovery furnace operated continuously throughout the day, after heating the furnace bath and forming the skull, the following mixture was gradually loaded into the furnace every 2 h: Briquette—5 kg; Coal—4 kg and Quartzite—1.9 kg.

**Table 3:** Study planning matrix.

No	Coded Variables		Natural Variables	
	X1	X2	Coal (C), kg	Quartzite (Q), kg
1	1	1	94.18	34.51
2	− 1	1	65.82	34.51
3	1	− 1	94.18	25.13
4	− 1	− 1	65.82	25.13
5	1.41	0	100	29.82
6	− 1.41	0	60	29.82
7	0	1.41	80	36.4
8	0	− 1.41	80	23.2
9–13	0	0	80	29.82

Main characteristics of the furnace bath: diameter 300 mm, depth 300 mm, V = 0.021 m^3^.

**Figure 2:**
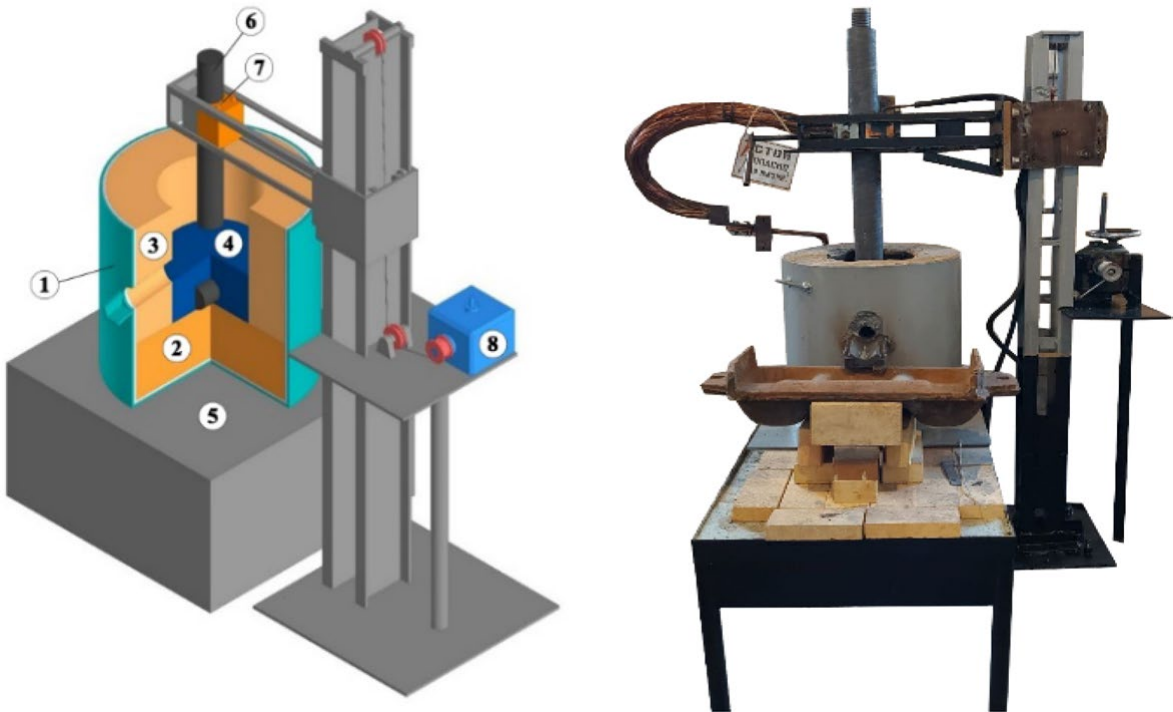
Laboratory ore-thermal electric furnace: I—3D model of an ore-thermal laboratory electric furnace, II—an ore-thermal laboratory electric furnace: 1—Furnace body, 2, 3—Refractory materials (brick), 4—Furnace hearth, 5—Lower electrode, 6—Electrode, 7—Electrode holder, 8—Electrode holder mechanism.

Laboratory tests for the smelting of ligature containing Fe-Si-Mn-Al using a manganese-containing briquette, high-ash coals of the Saryadyr deposit were carried out in a two-electrode ore-thermal electric furnace with a conductive hearth, and one electrode was coked in the hearth by the hearth mass, that is, the electric furnace has the structure similar to the Mige type electric furnace. The 3D model and the constructed ore-thermal laboratory electric furnace are shown in the Fig. 2^40–43^.

The main advantage of a laboratory ore-thermal electric furnace is lower energy and material consumption than a semi-industrial furnace with a capacity of 200–250 kVA, while it has the ability to solve the same technological problems.

The main characteristics of the furnace bath are: the diameter is 300mm, the depth is 300 mm, and the graphite electrode used has a diameter of 100 mm. Power transformer type OSZ-250/0.5 UHL4. Cooling—natural air. PBV (switching without excitation). Frequency—50 Hz. Insulation heat resistance class—F. Phase—1. Scheme and winding connection group—1/1–0. The transformer has seven voltage levels on the secondary side from 27.5 V to 71.3 V. In the laboratory ore-thermal furnace, according to the geometry of the bath and the diameter of the furnace electrodes, the following voltage levels are used on the secondary side of 27.5 V. The furnace bath is lined with fireclay bricks. The hearth of the furnace is packed with electrode mass, preheated to 100–120 °C. The surface of the hearth has a slope at an angle of 5°–7° in the direction of the tap hole to facilitate the release of the melt.

## Results and discussion

**Figure 3:**
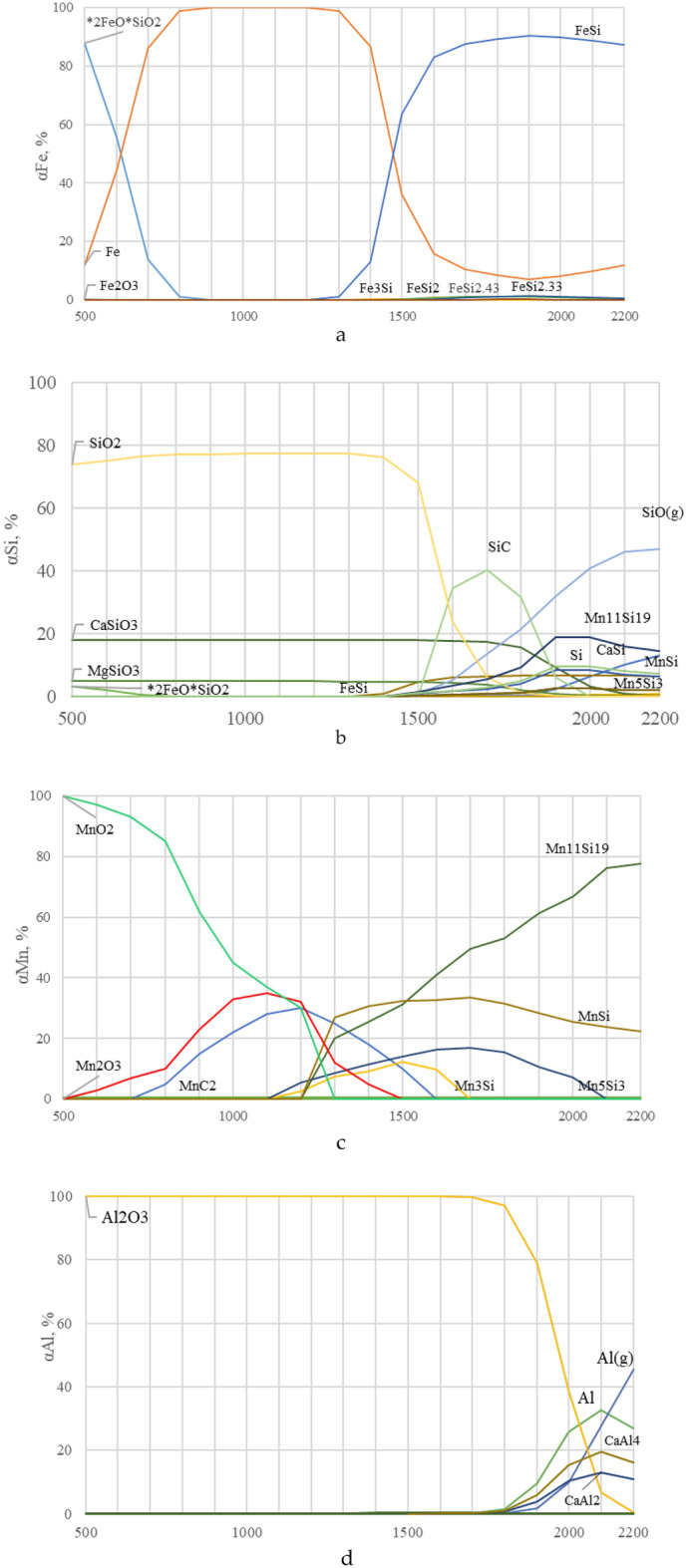
Effect of temperature on the equilibrium degree of distribution of element in the BCQ system: (**a**) iron; (**b**) silicon; (**c**) manganese; (**d**) aluminum.

On the Fig. 3 shows the effect of temperature on the quantitative (kg) distribution of iron, silicon, manganese and aluminum, and forming components, in the system under consideration, from a charge containing: 100 kg of briquette, 80 kg of coal and 29.8 kg of quartzite (BCQ—briquette-coal—quartzite). From the results obtained, it can be stated that iron occurs in the alloy in the form of FeSi, which begins to form at 1300 °C (Fig. 3a). Other iron silicides, Fe_3_Si, FeSi_2_, FeSi_2.43_, and FeSi_2.33_, are also formed in small amounts. Silicon, in addition to iron silicides, also occurs in the form of manganese silicides and in the form of free silicon (Fig. 3b).

According to the results of thermodynamic modeling, manganese-containing substances (Fig. 3c) under equilibrium conditions are in the form of manganese silicides—Mn_11_Si_19_, MnSi, Mn_5_Si_3_, Mn_3_Si. It should be noted that the bulk of manganese silicides is Mn_11_Si_19_. Aluminum-containing substances under equilibrium conditions are in the form of CaAl_2_, CaAl_4_ and Al. These substances begin to form at 1700–1800 °C (Fig. 3d).

**Figure 4:**
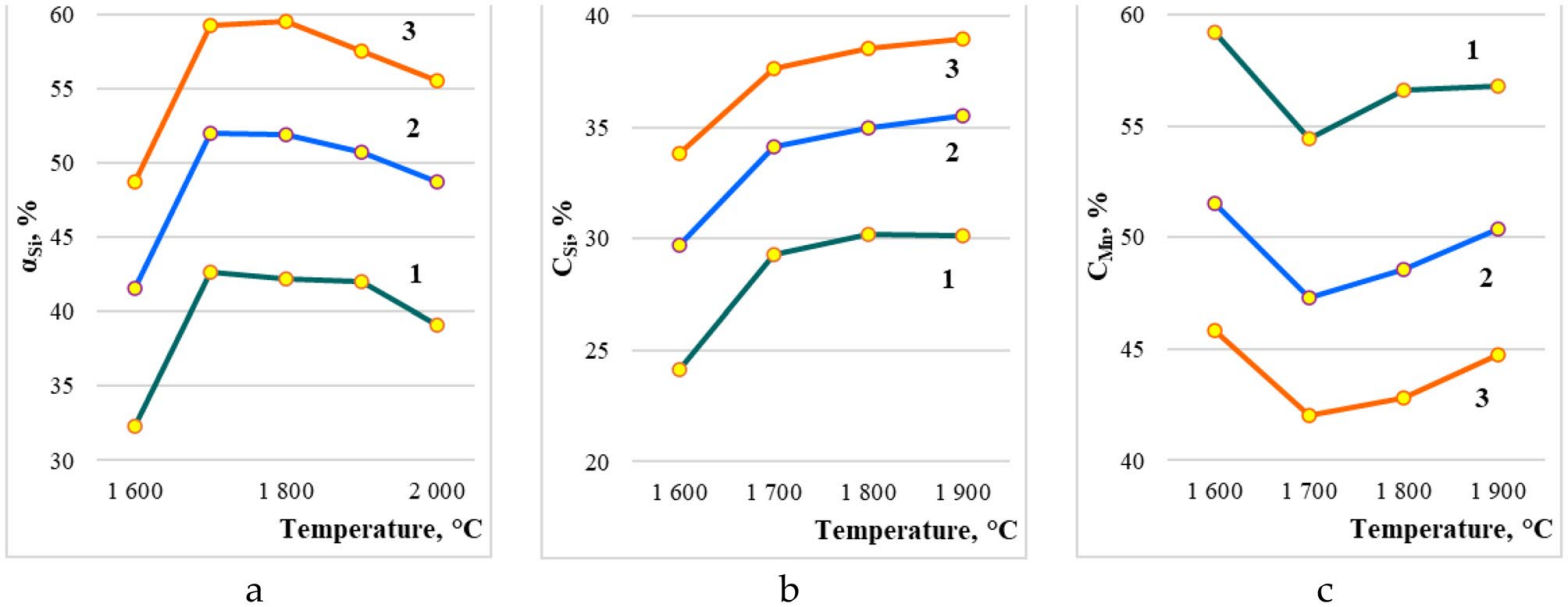
The influence of temperature and carbon on: (**a**) the extraction of silicon into the alloy; (**b**) the concentration of silicon; (**c**) manganese in the alloy.

On the Fig. 4. the effect of temperature, the amount of quartzite, coal on the equilibrium degree of silicon extraction into the alloy and the concentration of silicon and manganese in the alloy is shown. The degree of transition of Mn into the alloy is not shown, since almost all of the manganese passes into the alloy at a temperature of 1567 °C. From Fig. 4a it follows that with an increase in coal from 40 to 100%, the degree of transition of Si into the alloy increases. For example, at 1800 °C from 42.43 to 59.55%. Moreover, with an increase in temperature above 1700–1800 °C, the degree of extraction of silicon into the alloy somewhat decreases. Since, with increasing temperature, silicon, in the form of SiO gas, is sublimated (removed) from the furnace. The silicon concentration in the alloy also depends on the amount of coal. Thus, at 1800 °C, the Si concentration in the alloy increases by 27.4% (from 30.2 to 38.5%) (Fig. 4b). A more complex dependence of the effect of temperature on the concentration of manganese in the alloy. This dependence has a minimum at 1700 °C in the range of coal amount from 40 to 100%. With an increase in the coal charge, the concentration of Mn in the alloy decreases (due to the development of the silicon reduction process), for example, at 1800 °C from 56.6 to 48.84% (Fig. 4c).

**Figure 5:**
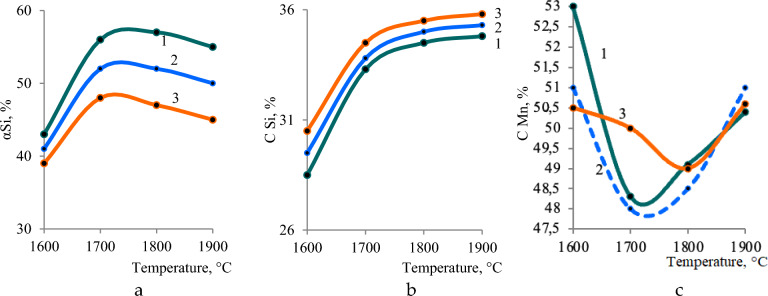
The effect of temperature and the amount of quartzite on the extraction of: (**a**) the extraction of silicon into the alloy; (**b**) the concentration of silicon; (**c**) manganese in the alloy.

**Figure 6:**
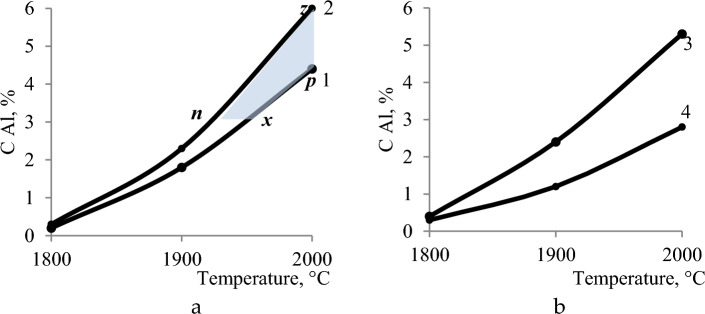
Effect of mineral quantity temperature on aluminum concentration in the alloy: (**a**) coal, (**b**) quartzite.

With a constant amount of coal of 28% (Fig. 5a) an increase in the amount of quartzite reduces the degree of extraction of silicon into the alloy, for example, at 1800 °C from 56.24% to 47.23%. Dependence αSi = f(T), has a maximum at 1800 °C. The silicon concentration in the alloy increases slightly with the increase in quartzite in the charge (Fig. 5b). A more complex dependence of the influence of temperature and quartzite on the concentration of manganese in the alloy. The maximum concentration of manganese is observed at 1600 °C, when the reduction of silicon is not yet developed. The minimum content of Mn is observed at 1700–1800 °C (Fig. 5c). Figure 6 shows that with an increase in the amount of coal, the concentration in the aluminum alloy increases, and with an increase in quartzite, it decreases. The maximum aluminum content in the alloy (6.1%) is observed at 2000 °C and 100 kg coal. At a constant amount of carbon, an increase in quartzite in the charge reduces the degree of extraction of silicon into the alloy, for example, at 1800 °C from 56.2 to 47.2%.

To determine the optimal parameters of the smelting process of the complex ligature, further studies were carried out by the method of planning experiments using rotatable plans of the second order (the Box-Hunter plan)^36^. The research planning matrix is shown in Table 3, and their results on the influence of the amount of coal and quartzite on the degree of transition of silicon into the alloy, the concentration of silicon and manganese in the alloy at temperatures of 1600–1900 °C are shown in (^44^, pp. 64–66).

Using the data from, we have obtained the following adequate regression equations (Table 4):

**Table 4:** Regression equations at temperatures of 1600–1900 °C.

No	T, °C	Regression equations
1	1600	CSi_(alloy)_ = − 12.552 + 0.571∙C + 0.526∙Q − 1.835∙10^−3^∙C^2^ + 4.738∙10^–3^∙Q^2^ − 1.308∙10^–3^∙C∙Q
2		CMn_(alloy)_ = 100.417 + 2.153∙10^–3^∙C − 0.389∙Q − 0.618∙C^2^ + 3∙10^–3^∙Q^2^ − 3.007∙10^–4^∙C∙Q
3		αSi_(alloy)_ = 3.832 + 0.736∙C − 0.234∙Q − 1.860∙10^–3^∙C^2^ + 5.910∙10^–4^∙Q^2^ − 1.202∙10^–3^∙C∙Q
4	1700	CSi_(alloy)_ = 8.587 + 0.367∙C + 0.083∙Q − 0.001∙C^2^ − 0.002∙Q^2^ − 0.001∙C∙Q
5		CMn_(alloy)_ = 89.993 − 0.312∙10^–3^∙C − 1.204∙Q + 4.426∙10^–4^∙C^2^ + 0.024∙Q^2^ − 0.002∙C∙Q
6		αSi_(alloy)_ = 19.6 + 0.783∙C − 0.469∙Q − 0.003∙C^2^ + 0.006∙Q^2^ − 0.003∙C∙Q
7	1800	CSi_(alloy)_ = 5.877 + 0.418∙C + 0.216∙Q − 0.002∙C^2^ + 0.005∙Q^2^ − 0.001∙C∙Q
8		CMn_(alloy)_ = 93.595 − 0.699∙C − 0.227∙Q − 0.003∙C^2^ + 0.009∙Q^2^ − 0.004∙10^–4^∙C∙Q
9		αSi_(alloy)_ = 25.6 + 0.718∙C − 0.625∙Q − 0.002∙C^2^ − 0.005∙Q^2^ + 0.003∙C∙Q
10	1900	CSi_(alloy)_ = 7.154 + 0.455∙C + 0.004∙Q − 0.002∙C^2^ − 0.004∙Q^2^ + 0.004∙C∙Q
11		CMn_(alloy)_ = 70.12 − 0.314∙C + 0.299∙Q + 5.645∙10^–4^∙C^2^ − 0.001∙Q^2^ − 0.002∙10^–4^∙C∙Q
12		αSi_(alloy)_ = 40.817 + 0.574∙C − 1.230∙Q − 0.002∙C^2^ − 0.002∙Q^2^ + 0.006∙C∙Q

*C coal, *Q* quartzite.

**Figure 7:**
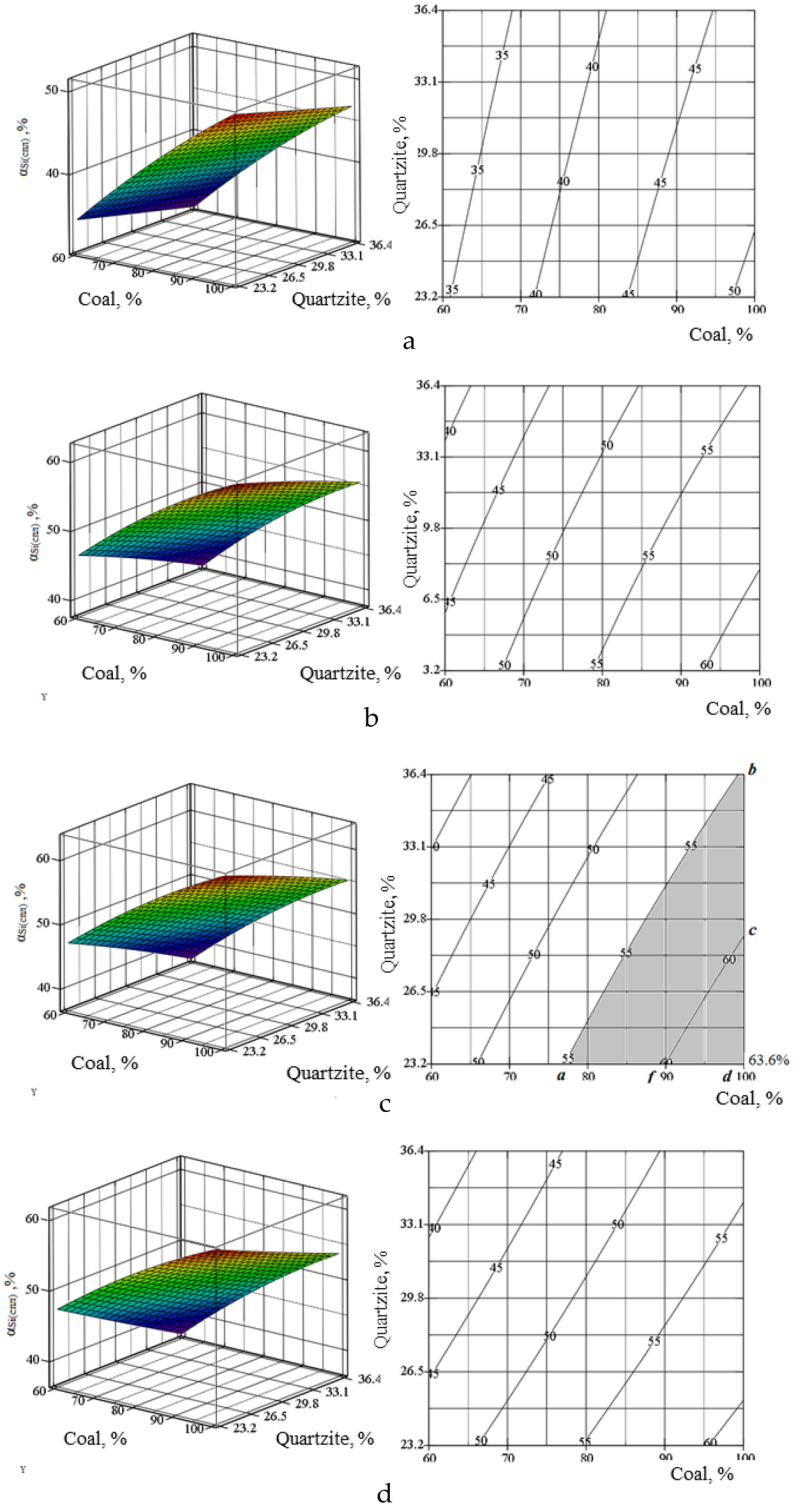
Volumetric and planar figures of the influence of the amount of coal and quartzite on the degree of extraction of silicon into the alloy: (**a**) 1600 °C, (**b**) 1700 °C, (**c**) 1800 °C, (**d**) 1900 °C.

**Figure 8:**
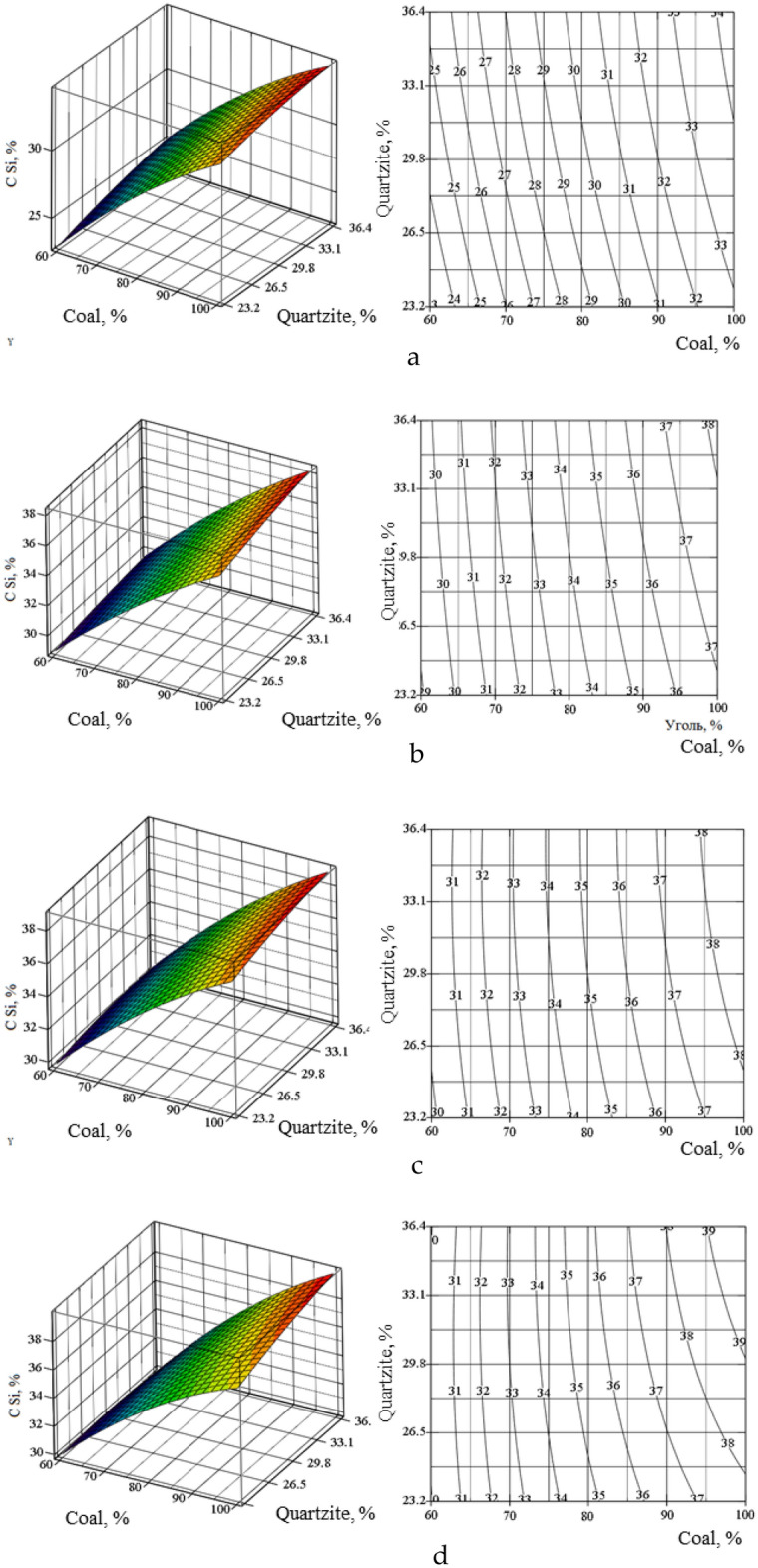
Volumetric and planar figures of the influence of the amount of coal and quartzite on the concentration of silicon in the alloy: (**a**) 1600 °C, (**b**) 1700 °C, (**c**) 1800 °C, (**d**) 1900 °C.

**Figure 9:**
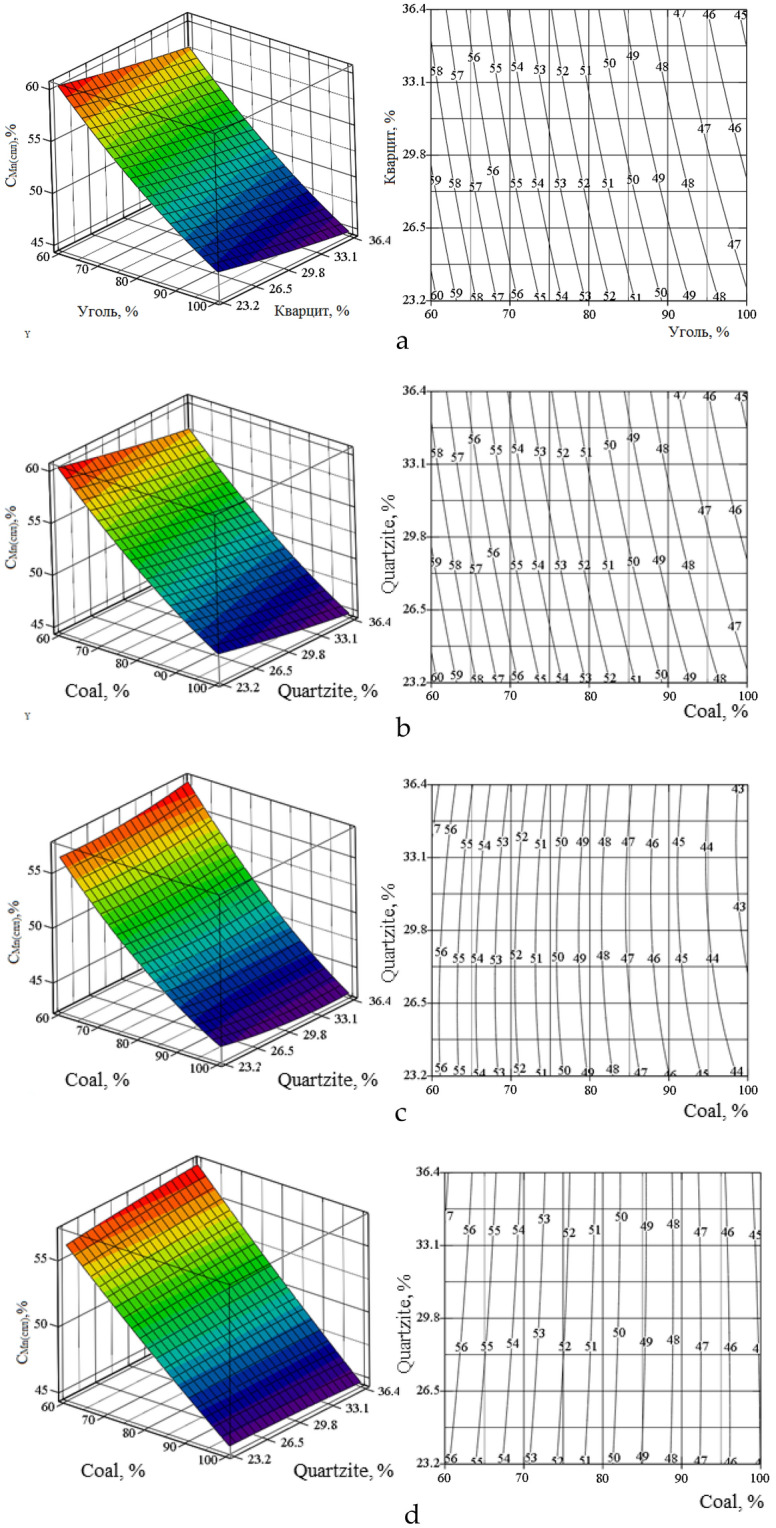
Volumetric (I) and planar (II) figures of the influence of the amount of coal and quartzite on the concentration of manganese in the alloy: (**a**) 1600 °C, (**b**) 1700 °C, (**c**) 1800 °C, (**d**) 1900 °C.

On the basis of the obtained equations, using the Mathcad program^34–37^, we constructed volumetric and planar figures of the change α_Si_(_alloy_), C_Mn_(_alloy_), C_Si_(_alloy_) in the temperature range 1600–1900 (Figs. 7, 8, 9). The degree of recovery of changes in silicon αSi(_alloy_), concentrations of manganese CMn(_alloy_) and silicon CSi(_alloy_) in the temperature range are indicated by Roman numerals a–d.

Figure 7a–d shows that in order to achieve the maximum extraction of silicon into the alloy, a temperature of 1800 °C and 100% amount of coal are required. From Fig. 8a–d it follows that in the region of 1700–1800 °C, the concentration of silicon in the alloy is 29–38.8%. According to this indicator, based on^12^, the alloy corresponds to the FeMnSi28 (Si = 28–30%) or MnC25 (Si > 25%) ferroalloy. However, according to the manganese content of 41.9–57.2% (Fig. 9a–d), the alloy does not meet the standard^12^, according to which the Mn content in ferrosilicomanganese should be at the level of 60–75%. Therefore, ferroalloys formed at 1700–1800 °C cannot be attributed to ferrosilicomanganese. They can only be attributed to ligatures. Figure 5c shows that in the system under consideration, manganese is extracted into the alloy more than silicon. Therefore, the optimal parameters must be set based on the maximum extraction of silicon into the alloy. Table 5 shows the technological parameters at αSi(spl) ≥ 55% and ≥ 60% and a temperature of 1800 °C.

**Table 5:** Values of technological parameters at boundary points at αSi_(spl)_ ≥ 55% and αSi_(spl)_ ≥ 60%.

Points in Fig. 6	Technological parameters					
	T, °C	Quartzite, %	Coal, %	αSi(sm), %	CSi(spl), %	CMn(spl), %
A	1800	23.2	73.7	55.0	33.9	50.3
B	1800	36.4	100.0	55.0	38.8	43.1
C	1800	28.9	100.0	60.0	38.3	43.2
D	1800	23.2	100.0	63.6	37.7	44.2
F	1800	23.2	100.0	60.0	36.2	46.0

It can be seen from Table 5 that when αSi(cm) is from 60 to 63.6% (fcd region), the Si content in the alloy is from 36.2 to 38.3% and Mn from 43.2 to 46.0% is possible when the amount quartzite is 23.2–28.9%, and coal from 90 to 100%. An alloy containing 50% Mn can be obtained at 1800 °C, 23.2% quartzite, 73.7% coal. while αSi(cm) = 55%, and CSi(cm) decreases to 33.9%.

To obtain a ligature that will contain aluminum, it is necessary to increase the process temperature (more than 1900 °C). So, from Fig. 6a it can be seen that the Al content in the alloy can be equal to 3.0–6.1% with 40–100% coal and 28% quartzite. Technological parameters of alloys with a high content of aluminum at the boundary points of the plane ***zpxn*** shown in Table 6. And when the quartzite content in the charge is 20 and 36% (Fig. 6b), the aluminum concentration contains 2.7–5.1%. Which does not provide the maximum concentration of aluminum in the alloy.

**Table 6:** The value of technological parameters at the boundary points of the area zpxn of Fig. 5a.

Points in Fig. 5 (I)	Technological parameters						
	T, °C	Quartzite, %	Coal, %	αSi_(sm)_, %	CSi_(spl)_, %	CMn_(spl)_, %	CAl_(spl)_, %
Z	2000	28.0	100	56.3	35.8	42.6	6.1
P	2000	28.0	40	42.1	27.6	55.9	4.4
X	1950	28.0	40	42.3	32.0	57.0	2.7
N	1950	28.0	100	56.6	38.8	45.3	2.7

For approbation and development of the technological scheme of production, a series of laboratory tests was carried out.Melting was carried out in an ore-thermal electric furnace with a capacity of 150 kVA in a continuous way by loading the charge in small portions as the top shrinks and with periodic release of the alloy every 2 h into cast-iron molds. At a constant consumption of a briquette (100 kg), 12 melt outlets were produced over the period, where the adjustment was carried out only for the consumption of high-ash coal: 60, 80 and 100 kg. Forneutralization of residual carbon used quartzite.The weighted average chemical composition of the Fe-Si-Mn-Al complex ligature is shown in Table 7.

**Table 7:** Chemical composition of the Fe-Si-Mn-Al complex ligature, %.

Consumption of high-ash coal, kg	Chemical composition, %			
	Fe	Si	Mn	Al
60	7.42	45.92	34.81	5.60
80	7.19	49.92	30.86	6.33
100	7.01	53.11	27.72	6.91

**Figure 10:**
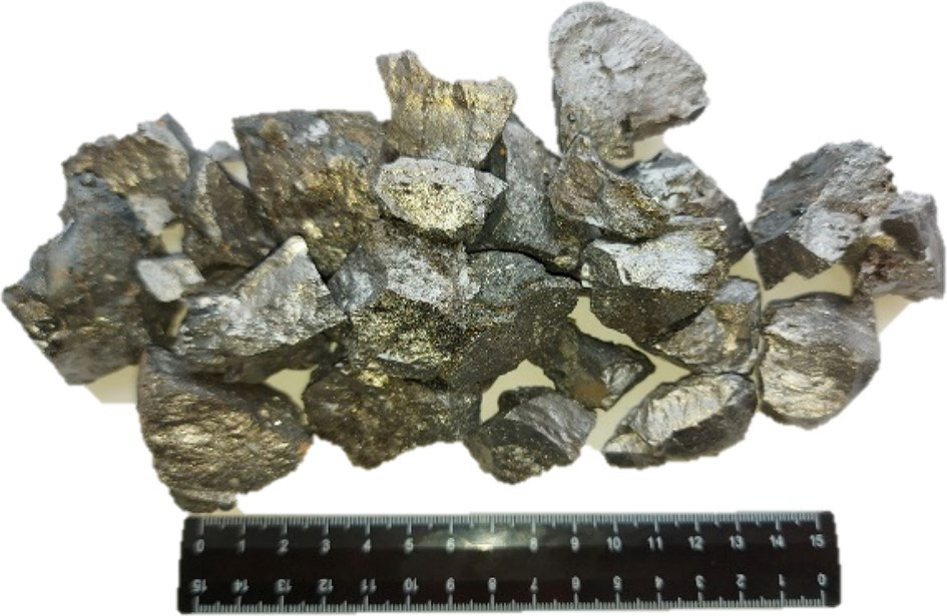
Complex ligature Fe-Si-Mn-Al obtained from manganese-containing briquette, high-ash coal of Saryadyr deposits and quartzite.

From Table 7 it can be seen that with an increase in the amount of coal, the concentration of silicon in the alloy noticeably increases. This is due to the fact that coal ash contains 67.05% SiO_2_. In addition, with an increase in the mass of coal in the charge, residual carbon increases, to neutralize which quartzite is introduced to form silicon. On the Fig. 10 shows a complex ligature obtained from 80 kg of high-ash coal, 100 kg of manganese-containing briquette and 39.38 kg of quartzite. The resulting alloy, according to the standards, according to the content of Si (45 ÷ 53) and Mn (27 ÷ 72) cannot be attributed to ferrosilicomanganese. Therefore, the resulting alloy can be confidently attributed to a complex ligature containing active elements (Si, Mn and Al), which makes it possible to effectively use it for the deoxidation of calm and semi-quiet steel grades.

## Conclusions

Based on the results of thermodynamic modeling under equilibrium conditions, the possibility of obtaining a complex ligature containing silicon, manganese and aluminum by a slag-free method from a manganese-containing briquette is shown. High-ash coal from the Saryadyr deposits was used as a reducing agent. According to the results of the calculation-theoretical method, modeling and laboratory experiments, the following was revealed:

- when interacting manganese-containing briquette with high-ash coalthe formation of an alloy consisting of iron and manganese silicides begins at a temperature of 1300 °C. The main phases of the alloy are FeSi and Mn_11_Si_19_, Fe_3_Si, FeSi_2_, FeSi_2.43_ and FeSi_2.33_, Mn_11_Si_19_, MnSi, Mn_5_Si_3_, Mn_3_Si. In the temperature range from 1700 to 1800 °C, aluminum-containing phases CaAl_2_, CaAl_4_ begin to form;
- an increase in coal charge from 40 to 100% increases the degree of transition of Si into the alloy; it increases from 42.43 to 59.55%. The silicon concentration in the alloy increases by 27.4% (from 30.2 to 38.5%);
- an increase in coal in the charge leads to a decrease in the concentration of Mn in the alloy from 56.6 to 48.84%, which is associated with the development of the silicon reduction process;
- an increase in quartzite in the charge has practically no effect on the extraction and concentration of silicon in the alloy, it significantly affects the concentration of manganese in the alloy, and reaches a maximum at 1600 °C, when the reduction of silicon is not yet developed. At a temperature of 1800 °C and a constant amount of coal in the charge of 28%, with an increase in quartzite, the degree of silicon extraction into the alloy decreases from 56.24% to 47.23%. With an increase in the amount of coal, the concentration of aluminum increases, and with an increase in quartzite, it decreases. The maximum aluminum content in the alloy (6.1%) is observed at 2000 °C and 100 kg of coal;
- the concentration of silicon in the alloy at temperatures of 1700–1800 °C is 29–38.8%. Based on this indicator, the alloy corresponds to FeMnSi_28_ (Si = 28–30%) or MnC_25_ (Si > 25%) ferroalloys. However, in terms of manganese content (41.9–57.2%), the alloy does not meet the standard, according to which the Mn content in ferrosilicomanganese should be 60–75%.

The obtained alloys in the temperature range of 1700–1800 °C using substandard raw materials (Table 1) in terms of chemical composition, do not belong to ferrosilicomanganese alloys, according to GOST 4756-91 (ISO 5447-80). They can be considered a new complex ligature with active elements of silicon, manganese and aluminum.for effective use in the deoxidation of calm and semi-quiet steel grades.

Approbation of the technology for obtaining a complex ligature Fe-Si-Mn-Al from manganese-containing briquettes and high-ash coals was carried out in a laboratory furnace with a capacity of 150 kVA and the optimal compositions of alloys and extraction of elements (in wt %) were established: Si—75–85, Al—60–70 and Mn—80–87. The experimental tests carried out indicate the fundamental possibility of smelting complex master alloys based on: Fe-Si-Mn-Al using the presented charge materials and alloys can be effectively usedduring deoxidation of calm and semi-calm steel grades.

As a result of the experiment, a complex ligature of Fe-Si-Mn-Al was obtained by the method of continuous melting in an ore-thermal electric furnace. The increase in the amount of high-ash coal in the charge led to an increase in the concentration of silicon in the alloy. Quartzite was used to neutralize residual carbon. The study confirms that with the interaction of manganese-containing briquettes with high-ash coal, it is possible to obtain a complex alloy without the slag method. Thermodynamic modeling shows that silicon, manganese and aluminum turn into an alloy from a temperature of 1300 °C. The increase in the amount of coal in the charge increases the degree of transition of silicon into the alloy and its concentration in the alloy, while the concentration of manganese decreases. The addition of quartzite to the charge has almost no effect on the concentration of silicon, but significantly affects the concentration of manganese in the alloy. The resulting alloys will be cheaper when introduced into the metallurgical cycle, as the main charge materials are high-ash coal and substandard manganese-containing materials are available at a low price. As the literary review shows, the competitiveness of the introduction of this alloy is not only related to the cost, but also to the improvement of the quality of steel after the deoxidation of steel with this alloy. It provides deep purification of steel from oxygen and non-metallic inclusions at the stage of deoxidation of calm and semi-calm steel grades, as opposed to the use of standard ferroalloys (ferromanganese, ferrosilicomanganese and ferrosilic) and ingot aluminum. The laboratory experiments carried out confirm the possibility of obtaining a complex Fe-Si-Mn-Al alloy from manganese-containing briquettes and high-ash coals.
